# A new species of the toothed toad *Oreolalax* (Anura, Megophryidae) from Sichuan Province, China

**DOI:** 10.3897/zookeys.929.49748

**Published:** 2020-04-22

**Authors:** Yinmeng Hou, Shengchao Shi, Daming Hu, Yue Deng, Jianping Jiang, Feng Xie, Bin Wang

**Affiliations:** 1 CAS Key Laboratory of Mountain Ecological Restoration and Bioresource Utilization and Ecological Restoration Biodiversity Conservation Key Laboratory of Sichuan Province, Chengdu Institute of Biology, Chinese Academy of Sciences, Chengdu 610041, China Chengdu Institute of Biology, Chinese Academy of Sciences Chengdu China; 2 Management Center of Sichuan White River National Nature Reserve, Pengzhou 611900, China Management Center of Sichuan White River National Nature Reserve Pengzhou China; 3 University of Chinese Academy of Sciences, Beijing 100049, China University of Chinese Academy of Sciences Beijing China

**Keywords:** Molecular phylogenetic analyses, morphology, southwest China, taxonomy

## Abstract

The toad genus *Oreolalax* is widely distributed in southwest China and northern Vietnam. A new species of the genus is described from Sichuan Province, China. Phylogenetic analyses based on the mitochondrial 12S rRNA and 16S rRNA gene sequences supported the new species as an independent clade clustered into the clade also containing *O. nanjiangensis* and *O. chuanbeiensis*. The new species can be distinguished from its congeners by a combination of the following characters: body size moderate (SVL 51.2–64.2 mm in males); head broad; tympanum hidden; interorbital region with dark triangular pattern; belly with marbling; male lacking spines on lip margin; spiny patches on chest small with thick sparse spines in male; nuptial spines thick and sparse; tibio-tarsal articulation reaching beyond nostril when leg stretched forward; toe webbing at base.

## Introduction

The toothed toad genus *Oreolalax* Myers & Leviton, 1962 belongs to Leptobrachiinae Dubois, 1980 of Megophryidae Bonaparte, 1850 (Amphibia, Anura). The genus currently contains 18 species (see list of Frost 2019), of which 17 species are known from southwestern China throughout the provinces of Sichuan, Shaanxi, Guizhou, Yunnan, Chongqing, Hunan, and Hubei (Fei et al. 2012, 2016), and one from northernmost Vietnam (Nguyen et al. 2013). Roy et al. (2018) noted one specimen of *Oreolalax* from northeastern India, but the photo of the specimen (Roy et al. 2018: fig. 1f) seems to fit the generic characters of the genus *Scutiger* Theobald, 1868 (according to keys in Fei et al. 2016). Toads of *Oreolalax* inhabit mountain forests at elevations between ca. 500 and 3300 m a.s.l. (Fei et al. 2009, 2012, 2016; Nguyen et al. 2013).

Although taxonomic assignments of the genus *Oreolalax* have been controversial for decades (Liu 1950; Dubois 1980, 1983, 1986; Huang 1991; Zhao and Adler 1993; Dubois and Ohler 1998; Delorme 2001; Wei et al. 2007, 2009; Fei et al. 1989, 1990, 2005, 2009), most phylogenetic studies indicated the genus as a monophyletic group (Xu et al. 1992; Fu et al. 2007; Wei et al. 2009; Pyron and Wiens 2011; Nguyen et al. 2013), and most recent taxonomic arrangements also regarded it as a distinct genus (e.g., Fei et al. 2012, 2016; Frost et al. 2019). As noted, the phylogenetic relationships between many species of the genus have been not resolved (Fu et al. 2007; Nguyen et al. 2013) although species of the genus in China had been divided into several species groups based on morphology (Fei et al. 2005, 2009).

Hengduan Mountains with the adjacent eastern mountains were suggested as the centre of origin and differentiation of the *Oreolalax* toads (Fei et al. 1989) in view of twelve species (66.7% of total number 18) being distributed in the narrow but long zone in the central southern part of Hengduan Mountains (Fei et al. 2012, 2016). As well, this region forms an important part of a biodiversity hotspot (Myers et al. 2000), and is expected to support underestimated species diversity. However, for two decades, no new species of *Oreolalax* has been reported in China, being much poorer on species diversity in contrast to the high species richness of the co-family genera *Megophrys* Kuhl & Van Hasselt, 1822 and *Leptobrachella* Smith, 1925 (e.g., Li et al. 2018; Zhang et al. 2017), and still contain dozens of cryptic species (Liu et al. 2018; Chen et al. 2016). Obviously, lacking deep investigation is the most significant obstacle for detecting cryptic species of *Oreolalax* in view that there has been no detailed taxonomic evaluation on its populations for two decades based on molecular phylogenetic data.

During the field surveys in 2018 in the White River National Nature Reserve, Pengzhou City, Sichuan Province (Prov.), China, we collected nine *Oreolalax* specimens. Our detailed morphological comparisons and molecular phylogenetic analyses indicate that the specimens should represent an undescribed species. Herein we describe it as a new species.

## Materials and methods

### Specimens

**Figure 1.**
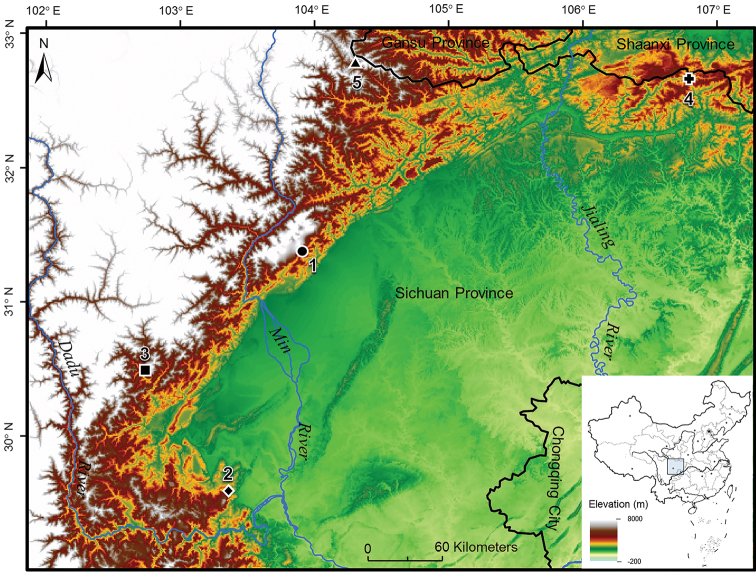
Localities for specimens used in this study. All localities are in Sichuan Province, China. Key: **1** Sichuan White River National Nature Reserve in Pengzhou City as the type locality of *Oreolalax longmenmontis* sp. nov.; **2** E mei Mountain as the common type locality of *O. major* and *O. omeimontis*; **3** Baoxing County as the type locality of *O. popei*, also as another sampling locality of *O. major*; **4** Guangwu Mountain in Nanjiang County as the type locality of *O. nanjiangensis*; **5** Pingwu County as the type locality of *O. chuanbeiensis*.

Three adult males and six tadpoles of *Oreolalax longmenmontis* sp. nov. were collected in May 2018 in the White River National Nature Reserve, Pengzhou City, Sichuan Prov., China (Fig. 1; Table 1, Suppl. materials 1, 2). For comparisons, some specimens of congeneric species were also collected in Sichuan Prov., China: eight *O. major* (Liu & Hu, 1960) from E’ mei Mountain (the type locality of the species) and Baoxing County (Co.), one *O. nanjiangensis* Fei & Ye, 1999 from Guangwu Mountain, Nanjiang Co., and four *O. omeimontis* (Liu & Hu, 1960) from E’mei Mountain (the type locality of the species; Fig. 1; Table 1, Suppl. materials 1, 2). After taking photographs, the toad and tadpole were euthanized using isoflurane, and the specimens were fixed and then preserved in 75% ethanol. Tissue samples were taken and preserved separately in 95% ethanol prior to fixation. Specimens were deposited in Chengdu Institute of Biology, Chinese Academy of Sciences (**CIB**, **CAS**).

**Table 1.** Sampling information and GenBank accession numbers for molecular samples used in this study.

ID	Species	Voucher number	Locality (County/City, Province, Country)	12S	16S
1	*Oreolalax longmenmontis* sp. nov.	CIB20180522001	Pengzhou, Sichuan, China	MN749799	MN688667
2	*Oreolalax longmenmontis* sp. nov.	CIB20180526001	Pengzhou, Sichuan, China	MN749802	MN688670
3	*Oreolalax longmenmontis* sp. nov.	CIB20180526002	Pengzhou, Sichuan, China	MN749803	MN688671
4	*Oreolalax longmenmontis* sp. nov.	CIB2018041501	Pengzhou, Sichuan, China	MN749798	MN688666
5	*Oreolalax longmenmontis* sp. nov.	CIB2018052201602	Pengzhou, Sichuan, China	MN749800	MN688668
6	*Oreolalax longmenmontis* sp. nov.	CIB2018052201603	Pengzhou, Sichuan, China	MN749801	MN688669
7	*Oreolalax longmenmontis* sp. nov.	CIB2018041301	Pengzhou, Sichuan, China	MN749795	MN688663
8	*Oreolalax longmenmontis* sp. nov.	CIB2018041302	Pengzhou, Sichuan, China	MN749796	MN688664
9	*Oreolalax longmenmontis* sp. nov.	CIB2018041303	Pengzhou, Sichuan, China	MN749797	MN688665
10	*Oreolalax chuanbeiensis*	CIB-ZYC074	Mao County, Sichuan, China	EF397266	EF397266
11	*Oreolalax chuanbeiensis*	DQR-Pingwu-001J	Ping Wu, Sichuan, China	/	EU180887
12	*Oreolalax nanjiangensis*	CIBSCNJNJ2006004	Nanjiang, Sichuan, China	MN749790	MN688658
13	*Oreolalax nanjiangensis*	CIB-XM804	NanJiang, Sichuan, China	EF397265	EF397265
14	*Oreolalax multipunctatus*	CIB2013wb091	Emei, Sichuan, China	NC_037382	NC_037382
15	*Oreolalax omeimontis*	CIBWWS180610018	Emei, Sichuan, China	MN749793	MN688661
16	*Oreolalax omeimontis*	CIBWWS180610022	Emei, Sichuan, China	MN749794	MN688662
17	*Oreolalax omeimontis*	CIBEMS18061203	Emei, Sichuan, China	MN749791	MN688659
18	*Oreolalax omeimontis*	CIBEMS18061205	Emei, Sichuan, China	MN749792	MN688660
19	*Oreolalax rhodostigmatus*	CIB-ZYCA 746	Da Fang, Guizhou, China	EF397248	EF397248
20	*Oreolalax xiangchengensis*	CIB-3LW008	Li Jiang, Yunnan, China	EF397250	EF397250
21	*Oreolalax jingdongensis*	IOZCAS2691	Jingdong, Yunnan, China	EF397255	EF397255
22	*Oreolalax liangbeiensis*	IOZCAS3796	Puxiong, Yuexi, Sichuan, China	EF397253	EF397253
23	*Oreolalax rugosus*	CIB-XM340	Shi Mian, Sichuan, China	EF397254	EF397254
24	*Oreolalax major*	CIB2019bx01	Baoxing, Sichuan, China	MN749782	MN688650
25	*Oreolalax major*	CIB2019bx02	Baoxing, Sichuan, China	MN749783	MN688651
26	*Oreolalax major*	CIB2019bx03	Baoxing, Sichuan, China	MN749784	MN688652
27	*Oreolalax major*	CIB2019bx04	Baoxing, Sichuan, China	MN749785	MN688653
28	*Oreolalax major*	CIB2019bx05	Baoxing, Sichuan, China	MN749786	MN688654
29	*Oreolalax major*	CIBEM1824	Emei, Sichuan, China	MN749787	MN688655
30	*Oreolalax major*	CIBEM1825	Emei, Sichuan, China	MN749788	MN688656
31	*Oreolalax major*	CIBEM1826	Emei, Sichuan, China	MN749789	MN688657
32	*Oreolalax schmidti*	ROM40457	Hongya, Sichuan, China	EF397257	EF397257
33	*Oreolalax pingii*	CIB-XM980	Xi Chang, Sichuan, China	EF397259	EF397259
34	*Oreolalax lichuanensis*	IZCASH30036	Lichuan, Hubei, China	EF544237	EF544237
35	*Oreolalax sterlingae*	IEBR A.2012.1	Sa Pa, Lao Cai, Vietnam	KC569979	KC569981
36	*Scutiger ningshanensis*	/	/	NC_031426	NC_031426
37	*Leptobrachella oshanensis*	CIB20050095	/	NC_020610	NC_020610

### Molecular data and phylogenetic analyses

Genomic DNA from each specimen collected in this work was extracted using a TIANamp Genomic DNA Kit by TIANGEN (BEIJING) BIOTECH, China. Fragments of the mitochondrial genes 12S rRNA and 16S rRNA were amplified. For 12S, the primers Fphe40L (5’-AAAGCACAGCACTGAAGAYGC) and 12S600H (5’-TTATCGATTATAGAACAGGCTCCTCT-3’) were used following Zhang et al. (2013), and for 16S, the primers P7 (5’-CGCCTGTTTACCAAAAACAT-3’) and P8 (5’-CCGGTCTGAACTCAGATCACGT-3’) were used following Simon et al. (1994). PCR amplification was performed in a reaction volume of 25 ul. Fragments were amplified under the following conditions: an initial denaturing step at 95 °C for 4 min; 36 cycles of denaturing at 95 °C for 30 s, annealing at 55 °C (for 16S)/52 °C (for 12S) for 30 s and extending at 72 °C for 60 s. Sequencing was conducted using an ABI3730 automated DNA sequencer in Shanghai DNA BioTechnologies Co., Ltd. (Shanghai, China). New sequences were deposited in GenBank (for accession numbers see Table 1).

For phylogenetic analyses, the available sequence data for all related species of *Oreolalax*, one *Scutiger ningshanensis* Fang, 1985, and one *Leptobrachella oshanensis* (Liu, 1950) were downloaded from GenBank especially for the topotypes of *Oreolalax* species (for accession numbers see Table 1). *Leptobrachella oshanensis* was used as the outgroup following Fu et al. (2007).

Sequences were assembled and aligned using the Clustalw options in BioEdit v. 7.0.9.0 (Hall 1999) with default settings. Alignments were checked by eye and modified manually if necessary. To avoid bias in alignments, GBLOCKS v. 0.91.b (Castresana 2000) was used to extract regions of defined sequence conservation from the length-variable 12S and 16S gene fragments with default settings. Non-sequenced fragments were regarded as missing loci. Two fragments were concatenated for the following phylogenetic analyses.

Phylogenetic analyses were conducted using maximum likelihood (ML) and Bayesian Inference (BI) methods, implemented in PhyML v. 3.0 (Guindon et al. 2010) and MrBayes v. 3.12 (Ronquist and Huelsenbeck 2003), respectively. Prior to phylogenetic analyses, 12S and 16S genes were defined as two partitions in the concatenated data, and jModelTest v. 2.1.2 (Darriba 2012) was used to select the best substitution model for each partition under the Bayesian Inference Criteria (BIC). The results suggested GTR + I + G model for all partitions. For the ML tree, branch supports were drawn from 10,000 nonparametric bootstrap replicates. In BI analyses, two runs each with four Markov chains were run for 40 million iterations with sampling every 1000 generations. The first 25% generations were removed as the “burn-in” stage followed by calculation of Bayesian posterior probabilities and the 50% majority-rule consensus of the post burn-in trees sampled at stationarity. Finally, genetic distance between *Oreolalax* species was calculated with the pairwise uncorrected *p*-distance model on 16S gene using MEGA v. 7 (Kumar et al. 2016).

### Morphological comparisons

Three adult males of *Oreolalax longmenmontis* sp. nov., four *O. nanjiangensis*, 13 *O. chuanbeiensis* Tian, 1983, and ten *O. popei* (Liu, 1947) were measured (Suppl. material 1). Sex of individuals was determined by presence of nuptial spines on finger and chest in males in breeding condition. The terminology and methods followed Fei et al. (2009) and Watters et al. (2016). Measurements were taken with a dial caliper to 0.1 mm. In total, 26 morphometric characters of adult specimen were measured:

**ED** eye diameter (distance from the anterior corner to the posterior corner of the eye)

**EN** eye to nostril distance (distance from anterior corner of the eye to the posterior margin of the nostril

**FIIIL** third finger length (distance from base to tip of finger III)

**FIIL** second finger length (distance from base to tip of finger II)

**FIL** first finger length (distance from base to tip of finger I)

**FIVL** fourth finger length (distance from base to tip of finger IV)

**FL** foot length (distance from tarsus to the tip of fourth toe)

**HDL** head length (distance from the tip of the snout to the articulation of jaw)

**HDW** maximum head width (greatest width between the left and right articulations of jaw)

**IND** internasal distance (minimum distance between the inner margins of the external nares)

**IOD** interorbital distance (minimum distance between the inner edges of the upper eyelids)

**LAL** length of lower arm and hand (distance from the elbow to the distal end of the finger III)

**LW** lower arm width (maximum width of the lower arm)

**NS** nostril–snout distance (distance from the tip of the snout to the naris)

**SL** snout length (distance from the tip of the snout to the anterior corner of the eye)

**SVL** snout-vent length (distance from the tip of the snout to the posterior edge of the vent)

**TFL** length of foot and tarsus (distance from the tibiotarsal articulation to the distal end of the toe IV)

**THL** thigh length (distance from vent to knee)

**TL** tibia length (distance from knee to tarsus)

**TOE1L** length of the first toe (distance from the metatarsal tubercle to the tip of toe I)

**TOE2L** length of the second toe (distance from the metatarsal tubercle to the tip of toe II)

**TOE3L** length of the third toe (distance from the metatarsal tubercle to the tip of toe III)

**TOE4L** length of the fourth toe (distance from the metatarsal tubercle to the tip of toe IV)

**TOE5L** length of the fourth toe (distance from the metatarsal tubercle to the tip of toe V)

**TW** maximal tibia width

**UEW** upper eyelid width (greatest width of the upper eyelid margins measured perpendicular to the anterior-posterior axis).

Six tadpoles (Suppl. material 2) were assigned to *O. longmenmontis* sp. nov. based on their phylogenetic positions very close to the adult specimens of the new species (see the results). Developmental stages were determined according to Gosner (1960). Twelve characters of tadpole specimen were measured:

**BH** maximum body height

**BW** maximum body width

**ED** maximum eye diameter

**IOD** interocular distance (minimum distance between eye)

**MW** mouth width (distance between two corners of mouth)

**SL** snout length (distance from the tip of the snout to the anterior corner of the eye)

**SS** snout to spiraculum (distance from spiraculum to the tip of the snout)

**SVL** snout-vent length

**TAH** tail height (maximum height between upper and lower edges of tail)

**TAL** tail length (distance from base of vent to the tip of tail)

**TBW** maximum width of tail base

**TOL** total length (distance from the tip of the snout to the tip of tail).

In order to reduce the impact of allometry, a corrected value from the ratio of each character to SVL was calculated and was log-transformed for subsequent morphometric analyses. Mann-Whitney *U* test was used to test the significance of differences on morphometric characters between species. The significance level was set at 0.05. Analyses were carried out in R.

We also compared morphological characters of *Oreolalax longmenmontis* sp. nov. with other *Oreolalax* species. Comparative morphological data were obtained from the literature for *O. chuanbeiensis* (Tian 1983; Fei et al. 2009, 2016), *O. granulosus* Fei, Ye & Chen, 1990 (Fei et al. 2009, 2016), *O. jingdongensis* Ma, Yang & Li, 1983 (Fei et al. 2009, 2016), *O. liangbeiensis* Liu & Fei, 1979 (Fei et al. 2009, 2016), *O. lichuanensis* Hu & Fei, 1979 (Fei et al. 2009, 2016), *O. major* (Fei et al. 2009, 2016), *O. multipunctatus* Wu, Zhao, Inger, and Shaffer, 1993 (Fei et al. 2009, 2016), *O. nanjiangensis* (Fei et al. 2009, 2016), *O. omeimontis* (Liu and Hu 1960; Fei et al. 2009, 2016), *O. pingii* (Liu, 1943) (Fei et al. 2009, 2016), *O. popei* (Liu 1947; Fei et al. 2009, 2016), *O. puxiongensis* Liu & Fei, 1979 (Fei et al. 2009, 2016), *O. rhodostigmatus* Hu & Fei, 1979 (Fei et al. 2009, 2016), *O. rugosus* (Liu, 1943) (Fei et al. 2009, 2016), *O. schmidti* (Liu, 1947) (Fei et al. 2009, 2016), *O. sterlingae* Nguyen, Phung, Le, Ziegler & Böhme, 2013 (Nguyen et al. 2013), *O. weigoldi* (Vogt, 1924) (Fei et al. 2009, 2016), and *O. xiangchengensis* Fei & Huang, 1983 (Fei et al. 2009, 2016). In addition, for comparison, we examined the holotype and/or topotype materials for *O. nanjiangensis*, *O. puxiongensis*, *O. liangbeiensis*, *O. multipunctatus*, *O. granulosus*, *O. chuanbeiensis*, *O. pingii*, *O. rugosus*, *O. schmidti*, *O. omeimontis*, *O. jingdongensis*, *O. lichuanensis*, *O. popei*, and *O. major* (Suppl. materials 1, 3).

## Results

**Figure 2.**
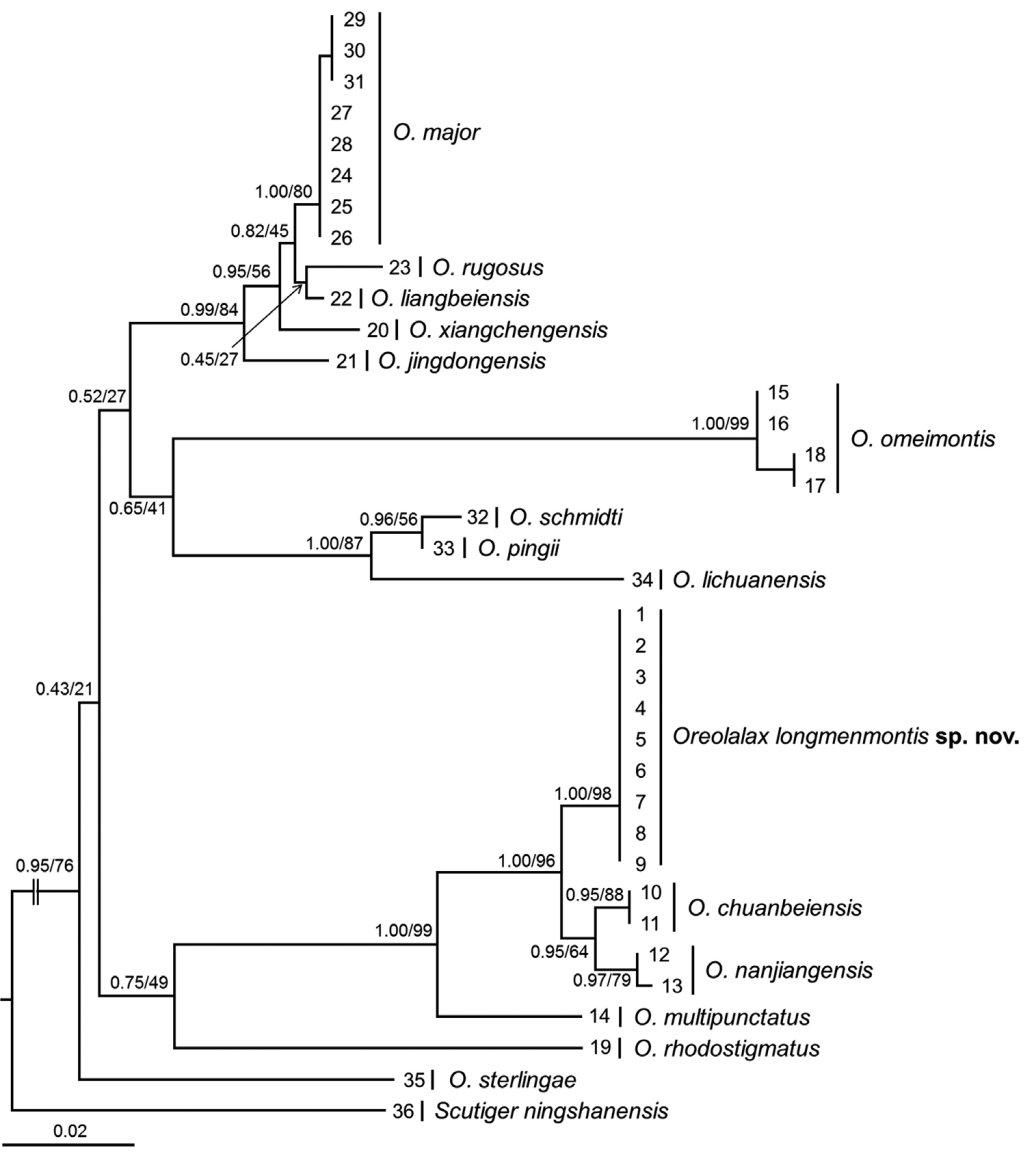
Maximum Likelihood tree based on the mitochondrial 12S and 16S gene sequences. Bayesian posterior probabilities from Bayesian Inference analyses/bootstrap supports from Maximum Likelihood analyses are labelled beside nodes. See information of samples 1–36 in Table 1.

Aligned sequence matrix of 12S+16S contained 896 bps. ML and BI trees presented almost consistent topology though relationships of some clades were unresolved (Fig. 2). The genus *Oreolalax* was strongly supported as a monophyletic group. All nine specimens of *O. longmenmontis* sp. nov. were clustered into one clade, which was independently clustered into a clade also containing *O. nanjiangensis* and *O. chuanbeiensis*. Genetic distance on 16S gene with uncorrected *p*-distance model between the nine specimens of *O. longmenmontis* was zero. Genetic distance between *O. longmenmontis* and its most closely related species *O. nanjiangensis* and *O. chuanbeiensis* were 1.4% and 1.6%, respectively, being higher than that between many pairs of species (Table 2), for example, *O. major* vs. *O. xiangchengensis* (0.6%), *O. rugosus* vs. *O. xiangchengensis* (1.3%), *O. rugosus* vs. *O. major* (1.0%), and *O. schmidti* vs. *O. pingii* (0.4%).

**Table 2.** Uncorrected *p*-distance between *Oreolalax* species of the 16S rRNA gene. Mean values of genetic distance are given in the lower half of the table.

	1	2	3	4	5	6	7	8	9	10	11	12	13	14
1. *Oreolalax longmenmontis* sp. nov.														
*2. Oreolalax nanjiangensis*	0.014													
*3. Oreolalax chuanbeiensis*	0.016	0.006												
*4. Oreolalax multipunctatus*	0.029	0.027	0.028											
*5. Oreolalax omeimontis*	0.085	0.093	0.091	0.087										
*6. Oreolalax rhodostigmatus*	0.065	0.065	0.066	0.059	0.096									
*7. Oreolalax xiangchengensis*	0.063	0.057	0.059	0.057	0.092	0.049								
*8. Oreolalax jingdongensis*	0.064	0.055	0.059	0.057	0.090	0.059	0.025							
*9. Oreolalax liangbeiensis*	0.063	0.056	0.058	0.056	0.088	0.049	0.007	0.018						
*10. Oreolalax rugosus*	0.057	0.056	0.058	0.055	0.087	0.047	0.013	0.025	0.011					
*11. Oreolalax major*	0.056	0.053	0.055	0.050	0.083	0.045	0.006	0.018	0.000	0.010				
*12. Oreolalax schmidti*	0.087	0.085	0.083	0.077	0.103	0.068	0.060	0.070	0.058	0.062	0.055			
*13. Oreolalax pingii*	0.080	0.079	0.076	0.069	0.097	0.061	0.055	0.066	0.054	0.055	0.049	0.004		
*14. Oreolalax lichuanensis*	0.095	0.089	0.086	0.084	0.113	0.080	0.066	0.082	0.069	0.069	0.067	0.032	0.032	
*15. Oreolalax sterlingae*	0.070	0.072	0.069	0.062	0.092	0.071	0.059	0.064	0.058	0.049	0.052	0.068	0.063	0.080

Mann-Whitney *U* tests indicated that *Oreolalax longmenmontis* was significantly different from *O. chuanbeiensis*, *O. nanjiangensis*, and *O. popei* on many morphometric characters (all P-values < 0.05; Table 3). *Oreolalax longmenmontis* could also be distinguished from its congeners based on morphological descriptions from the literature and from our examinations of specimens (Suppl. materials 1, 3). More detailed descriptions of results from morphological comparisons between the new taxon and its congeners are presented in the following sections.

**Table 3.** Morphometric comparisons between *Oreolalax longmenmontis* sp. nov. and its relatives. P-value from Mann-Whitney U test between the new species and each relative. The significant level is 0.05. See abbreviations for characters in the Materials and methods section.

Characters	*Oreolalax longmenmontis* sp. nov.		*Oreolalax popei*			*Oreolalax nanjiangensis*			*Oreolalax chuanbeiensis*		
	Male (*N* = 3)		Male (*N* = 10)		P-value	Male (*N* = 4)		P-value	Male (*N* = 13)		P-value
	Mean ± SD	Range	Mean ± SD	Range		Mean ± SD	Range		Mean ± SD	Range	
SVL	56.8 ± 6.7	51.2–64.2	64.3 ± 2.7	59.3–68.4	/	53.7 ± 1.6	51.4–55.0	/	50.9 ± 2.6	46.7–56.0	/
HDL	18.5 ± 1.9	17.2–20.6	24.0 ± 0.9	22.4–24.9	0.007	21.3 ± 0.4	21–21.8	0.057	17.9 ± 0.6	17.1–19.1	0.007
HDW	21.2 ± 2.1	19.1–23.3	23.9 ± 1.0	21.9–24.9	0.811	19.8 ± 0.9	19.1–21	0.857	17.7 ± 0.5	17.2–19.2	0.025
SL	8.2 ± 1.1	7.3–9.5	9.8 ± 0.4	9.0–10.5	0.014	8.1 ± 0.3	7.8–8.5	0.229	7.8 ± 0.4	7.1–8.4	0.014
NS	4.3 ± 0.4	3.8–4.6	4.6 ± 0.3	4.2–5.3	0.469	4.3 ± 0.2	4.1–4.5	0.114	4.3 ± 0.3	3.8–4.7	0.025
EN	4.0 ± 0.4	3.6–4.3	4.6 ± 0.3	4.1–5.3	0.692	3.7 ± 0.4	3.3–4.1	0.629	3.6 ± 0.2	3.2–4.0	0.800
IND	4.1 ± 0.4	3.7–4.5	4.7 ± 0.3	4.0–5.1	0.469	4.1 ± 0.1	3.9–4.2	0.114	4.7 ± 0.2	4.1–4.9	0.004
IOD	5.9 ± 0.4	5.5–6.2	6.4 ± 0.4	5.6–6.9	0.371	5.0 ± 0.5	4.3–5.4	0.229	5.8 ± 0.2	5.5–6.2	0.082
ED	6.1 ± 0.3	5.8–6.4	6.4 ± 0.4	5.5–6.8	0.112	5.0 ± 0.3	4.5–5.3	0.114	5.0 ± 0.2	4.6–5.5	0.189
UEW	5.3 ± 0.6	4.6–5.6	6.0 ± 0.2	5.6–6.3	0.573	5.0 ± 0.8	4.5–6.2	1.000	4.2 ± 0.2	3.9–4.5	0.014
LW	5.7 ± 0.7	5.0–6.3	6.5 ± 0.7	5.3–7.3	1.000	5.8 ± 0.5	5.1–6.3	0.229	5.7 ± 0.5	4.9–6.7	0.039
LAL	29.5 ± 1.6	28–31.2	30.2 ± 1.3	28.5–32.6	0.014	26.3 ± 1.2	24.5–27.2	0.229	26.5 ± 0.7	24.9–27.5	0.704
THL	29.8 ± 2.3	27.2–31.7	32.2 ± 1.4	30.1–34.3	0.161	27.4 ± 1.6	25.6–29.2	0.629	26.0 ± 1.0	23.6–27.3	0.364
TL	29.5 ± 3.5	25.7–32.8	31.4 ± 1.2	29.1–33.2	0.014	26.2 ± 0.9	25.1–27.4	0.114	25.1 ± 1.0	23–26.6	0.111
TW	6.5 ± 1.0	5.4–7.3	7.1 ± 0.5	6.5–7.8	1.000	6.0 ± 0.2	5.7–6.2	0.629	6.4 ± 0.9	4.4–7.4	0.439
TFL	44.1 ± 4.2	39.6–47.9	47.1 ± 1.7	44.6–49.7	0.770	39.0 ± 1.2	38.1–40.5	0.114	38.3 ± 1.3	36.0–40.5	0.189
FL	28.3 ± 3.3	25.4–31.9	31.4 ± 1.1	29.9–33.1	0.281	28.1 ± 1.7	26.5–30.0	0.114	25.8 ± 1.1	24.4–27.3	0.704

Based on the molecular and morphological differences, the specimens from the Sichuan White River National Nature Reserve, Sichuan Prov., China represent an new species which is described as follows.

### Oreolalax longmenmontis sp. nov.

Taxon classificationAnimaliaAnuraMegophryidae

88A4AA5B-28B5-59C0-A2C4-DBA12D5D4DEF

http://zoobank.org/9D057EEA-C908-41EF-B6EF-6A2FED872431

**Figure 3.**
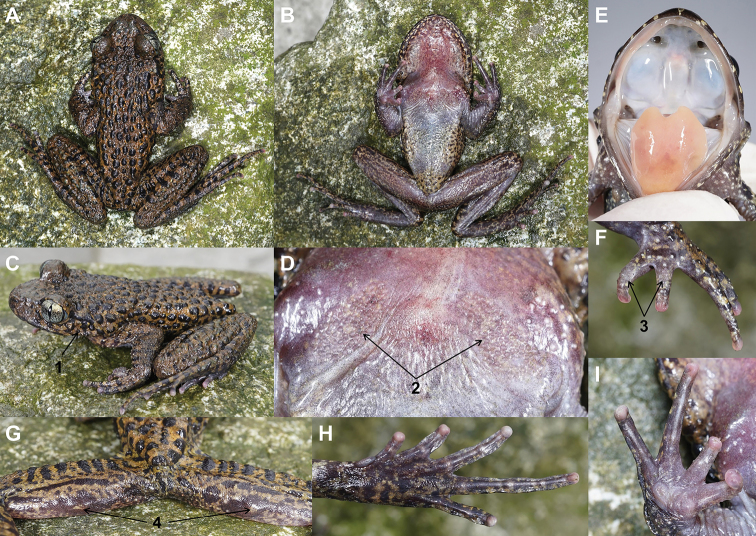
Photos of the holotype CIB20180522001 of *Oreolalax longmenmontis* sp. nov. in life **A** dorsal view **B** ventral view **C** lateral view **D** view of chest **E** view of oral cavity **E** dorsal view of fingers **G** view of femoris posterior **H** ventral view of foot **I** ventral view of hand. Key: 1 indicates tympanum hidden; 2 denotes a pair of spinal patches with large and sparse spines on chest; 3 denotes nuptial spines on the dorsal surface of fingers I and II; 4 denotes two small posterior femoral glands.

**Figure 4.**
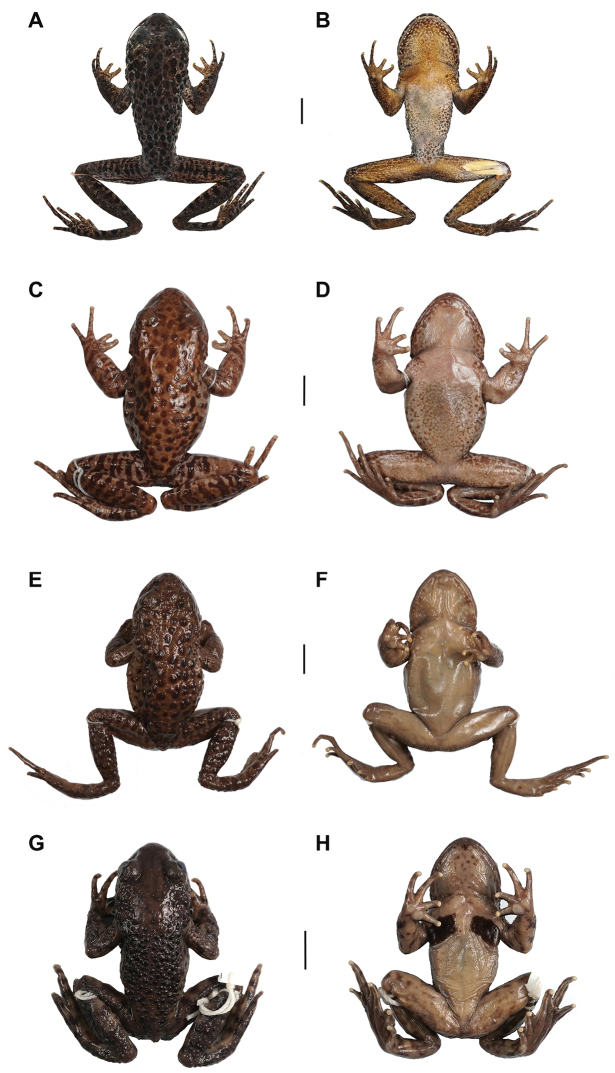
Specimen photos of *Oreolalax longmenmontis* sp. nov. and its relative species **A, B** dorsal and ventral view of the holotype CIB20180522001 of *Oreolalax longmenmontis* sp. nov. **C, D** dorsal and ventral view of the topotype CIB25142 of *O. popei***E, F** dorsal and ventral view of the topotype CIB89700 of *O. nanjiangensis***G, H** dorsal and ventral view of the topotype CIB89682 of *O. chuanbeiensis*. Scale bar: equal to 10 mm.

**Figure 5.**
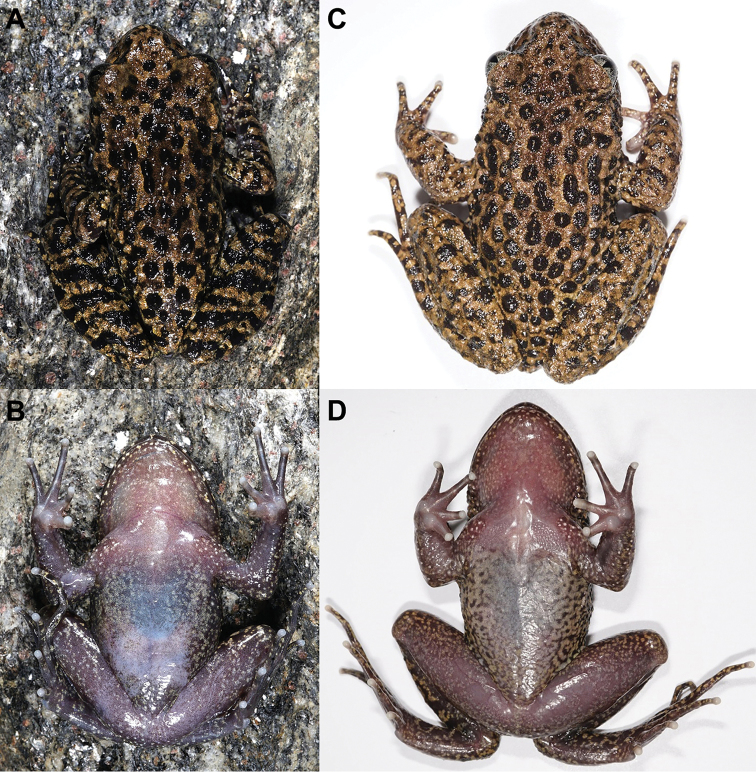
Colour variations in *Oreolalax longmenmontis* sp. nov. **A, B** dorsal and ventral view of the paratype CIB20180527002 **C, D** dorsal and ventral view of the paratype CIB20180526001.

**Figure 6.**
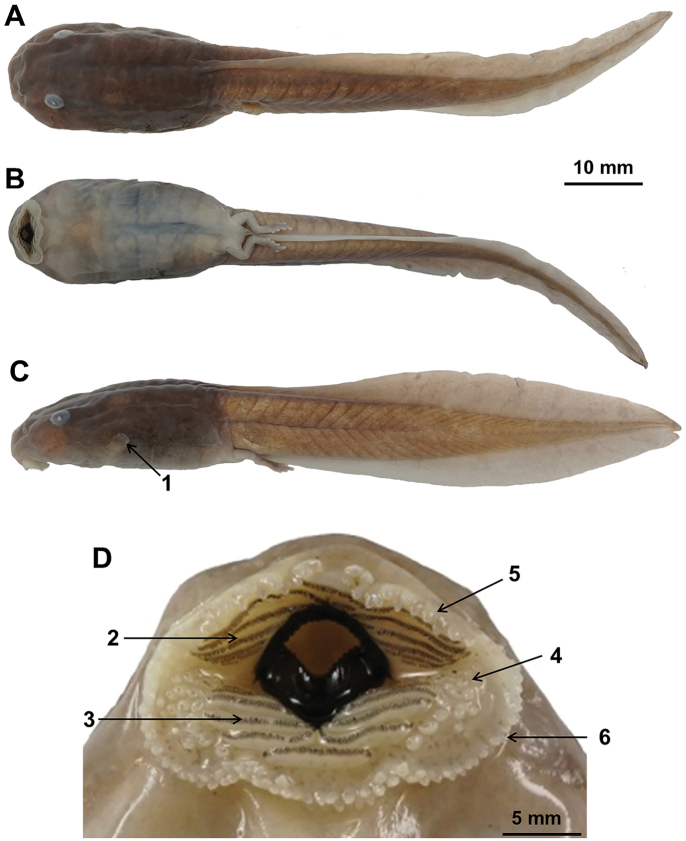
The tadpole specimen CIB2018041501 of *Oreolalax longmenmontis* sp. nov. **A** dorsal view **B** lateral view **C** ventral view **D** mouth structure. Key: 1 spiracle; 2 upper keratodonts; 3 lower keratodonts; 4 additional tubercles at the angles of mouth; 5 labial papillae on upper lips; 6 labial papillae on lower lips.

Figures 3 , 4A, B , 5 , 6 ; Tables 1 , 2 , Suppl. materials 1 , 2

#### Holotype.

CIB20180522001, adult male (Fig. 3), from Xia Jia Gou (31.293360N, 103.866190E, ca. 1335 m a. s. l.), White River National Nature Reserve, Pengzhou City, Sichuan Province, China, collected by SC Shi on 26 May 2018.

#### Paratype.

Two adult males collected from the same place of the holotype. Specimen CIB20180526001 collected by SC Shi on 26 May 2018; CIB20180527002 collected by B Wang on 27 May 2018.

#### Diagnosis.

*Oreolalax longmenmontis* sp. nov. is assigned to the genus *Oreolalax* by its molecular phylogenetic position and the following morphological characters: the maxillary teeth prominent; back rough scattered with large warts, covered with oval black spots; pupil vertical; tongue oval, notched posteriorly; femoral glands prominent; pectoral and axillary gland present in males in breeding season; inner two fingers with black nuptial spines in males in breeding season.

*Oreolalax longmenmontis* could be distinguished from its congeners by a combination of the following characters: body size moderate (SVL 51.2–64.2 mm in males); head broad; tympanum hidden; interorbital region with dark triangular pattern; belly with marbling; male lacking spines on lip margin; spiny patches on chest small with thick and sparse spines in male; nuptial spines thick and sparse; tibio-tarsal articulation reaching beyond nostril when leg stretched forward; toe webbing at base.

#### Description of holotype.

Measurements in mm. Body size medium, SVL 64.2; body relatively slender and flat; head wider than long (HDW/HDL ratio 1.14); snout rounded in dorsal and lateral views, slightly projecting beyond lower jaw; maxillary teeth present; vomerine range absent; eye large (ED 6.3), shorter than snout length (SL 9.5); pupil vertical; interorbital region flat; tympanum hidden; vocal sac absent; supratympanic fold significant; tongue longer than wide, free at the back, notched posteriorly; nostril oval, internarial distance (IND 4.5) shorter than upper eyelid (UEW 5.6), shorter than interorbital distance (IOD 6.2); nostril slightly closer to eye (EN 4.3) than to tip of the snout (NS 4.6).

Forelimbs moderately long and strong, length (LAL 31.2) approximately half of SVL; relative finger lengths: II < I < IV < III; fingers slender, distinct longitudinal ridges under fingers III and IV; finger tips rounded, two metacarpal tubercles oval, inner larger than outer; nuptial spines large and sparse on dorsal surface of fingers I and II.

Hindlimbs flat; toe webbing rudimentary, with narrow dermal fringes, distinct dermal ridges present under five toes; tibiotarsal articulation reaching beyond nostril when leg stretched forward; thigh length (THL 31.7) slightly shorter than tibia length (TL 32.8); foot length (FL 31.9) almost equal with thigh; relative toe lengths: I < II < V < III < IV; tips of toes rounded; subarticular tubercles distinct; inner metatarsal tubercle elliptical and narrow (IML 2.7), no outer metatarsal tubercle.

Rough skin on the back, lateral limb surfaces, large scattered tubercles with oval black spot; forehead and upper lip with scatted small tubercles; upper jaw protrudes slightly from lower jaw; supratympanic fold distinct, from posterior canthus above base of upper arm, mostly covered with dark spots. A pair of spinal patches small, present on chest, with relatively large and sparse spines; axillary glands small; posterior femoral gland small present. The backs of limbs with scatted differently sized tubercles; the forelimbs and hindlimbs have black longitudinal stripes, the hindlimbs are covered with medium-sized wart granules, the forelimbs are covered with many small white warty granules. Skin smooth on throat, belly, and ventral sides of the limbs.

#### Colouration of the holotype in life.

In life, body dark brown dorsally, with large tubercles, tubercular region with scattered black, oval-shaped markings, tongue orange-yellow, limb surfaces dark brown, scattered with different sizes of white tubercles; five or six faint transverse black stripes on the dorsal surface of the forearm; most parts of supratympanic line covered with black spots; belly interlaced with two colours: flesh red and greyish-white with some black speckles, throat and anterior chest are darker than belly. Back of posterior limbs with nine or ten black stripes; finger and toe tips flesh-pink. Arms and fingers covered in many scattered small white tubercles. Dorsal surfaces of head and hind limbs scattered with black medium-sized tubercles; upper lip barred with yellowish brown and black spots; iris bicolored; slightly beige above, silver below, with black reticulations throughout. Posterior femoral glands yellow-brown. Nuptial spines light grey, chest spiny patches flesh-pink. Outer metacarpals grey-pink, inner metatarsals brown.

#### Preserved holotype colouration.

In preservative (75% ethanol), the dorsal and lateral surfaces are dark brown; throat and anterior chest brown, belly grey, dark markings are evident on the abdomen and throat; the forelimbs and ventral surface of the thigh are brown, Inner and outer metacarpals brown; tongue creamy white; the colour of dorsal spots and stripes on limbs and posterior femoral glands become more conspicuous; the spiny patches become flat and indistinct (Fig. 4A, B).

#### Variations.

The two paratypes CIB20180526001 (Fig. 5A, B) and CIB20180527002 (Fig. 5C, D) are smaller than holotype on body size (Table 2, Suppl. material 1). The colour of paratypes is brown, lighter than holotype. The arrangement and shape of the large tubercles on the dorsal surface are more irregular than of the holotype. The paratype CIB20180526001 has fewer abdominal streaks than the holotype. The paratype CIB20180527002 has fewer markings at the meeting of thighs to abdomen and more markings in the posterior abdomen than holotype. Iris colour also varies between individuals: the holotype is light blue-green, CIB20180526001 is yellowish orange, and CIB20180527002 is orange.

#### Secondary sexual characteristics.

In breeding males, a pair of small spiny patches on chest, nuptial spines thick and sparse on dorsal surface of fingers I and II (Fig. 3D, H).

#### Tadpoles.

Measurements in mm. Differences in measurements are shown in Suppl. material 2. Character description is based on the preserved tadpole specimen CIB2018041501 (Fig. 6). Stage 37. Labial tooth row formula: 1:5+5/5+5:1; body dark brown in the back and lateral view, creamy white in the ventral; tail light brown; snout rounded; eye positioned dorsolateral; the opening of the spiracle single in the lateral, without a free distal tube; tail end blunt; caudal fin light and broad.

#### Comparisons.

By having a relatively larger body (SVL 51.2–64.2 mm in males; *N* = 3), *Oreolalax longmenmontis* sp. nov. differs from *O. pingii* (*N* = 20), *O. puxiongensis* (*N* = 20), *O. schmidti* (*N* = 30), *O. sterlingae* (*N* = 1), *O. multipunctatus* (*N* = 4), and *O. xiangchengensis* (*N* = 10) (vs. SVL < 51.0 mm in the latter).

By head wider than long, *O. longmenmontis* differs from *O. multipunctatus*, *O. rhodostigmatus*, and *O. schmidti* (vs. head longer than wide in the latter).

By the hidden tympanum, *Oreolalax longmenmontis* differs from *O. granulosus*, *O. jingdongensis*, *O. xiangchengensis*, and *O. rugosus* (tympanum absent in the latter) and differs from *O. rhodostigmatus* (tympanum rather visible).

By lacking spines on lip margin in male, *Oreolalax longmenmontis* sp. nov. differs from *O. sterlingae*, *O. granulosus*, *O. jingdongensis*, *O. liangbeiensis*, *O. lichuanensis*, *O. major*, *O. multipunctatus*, *O. omeimontis*, *O. pingii*, *O. puxiongensis*, *O. rugosus*, *O. schmidti*, *O. weigoldi*, and *O. xiangchengensis* (vs. spines on lip margin visible in male in the latter).

By vocal sac absent, *Oreolalax longmenmontis* differs from *O. omeimontis* (vs. vocal sac present in male in the latter).

By interorbital region with dark triangular pattern, *Oreolalax longmenmontis* differs from *O. multipunctatus*, *O. granulosus*, *O. major*, *O. liangbeiensis*, *O. lichuanensis*, *O. pingii*, *O. rhodostigmatus*, *O. rugosus*, *O. weigoldi*, *O. sterlingae*, and *O. xiangchengensis* (vs. without in the latter).

By spiny patches on chest small in male, *Oreolalax longmenmontis* differs from *O. granulosus*, *O. liangbeiensis*, *O. major*, *O. omeimontis*, *O. pingii*, *O. rhodostigmatus*, *O. jingdongensis*, *O. weigoldi*, and *O. xiangchengensis* (vs. large in the latter).

By spines on spiny patches on chest thick and sparse in male, *Oreolalax longmenmontis* differs from *O. omeimontis*, *O. granulosus*, *O. major*, *O. liangbeiensis*, *O. pingii*, *O. puxiongensis*, *O. rugosus*, *O. schmidti*, *O. sterlingae*, and *O. xiangchengensis* (vs. spines thin and fine in the latter).

By nuptial spines on fingers thick and sparse, *Oreolalax longmenmontis* differs from *O. sterlingae*, *O. omeimontis*, *O. granulosus*, *O. liangbeiensis*, *O. major*, *O. rugosus*, *O. schmidti*, *O. pingii*, *O. puxiongensis*, and *O. xiangchengensis* (vs. thin and fine in the latter).

By tibio-tarsal articulation reaching beyond nostril when leg stretched forward, *Oreolalax longmenmontis* differs from *O. omeimontis*, *O. multipunctatus*, *O. granulosus*, *O. major*, *O. liangbeiensis*, *O. lichuanensis*, *O. pingii*, *O. puxiongensis*, *O. rhodostigmatus*, and *O. rugosus* (vs. reaching up to the posterior corner of eye in the latter).

By toe webbing at base, *Oreolalax longmenmontis* sp. nov. differs from *O. granulosus*, *O. jingdongensis*, *O. liangbeiensis*, *O. major*, *O. rugosus*, *O. weigoldi*, and *O. xiangchengensis* (vs. toe IV at least 1/4 webbed in the latter).

By belly with marble spots, *Oreolalax longmenmontis* differs from *O. omeimontis*, *O. liangbeiensis*, *O. pingii*, *O. rhodostigmatus*, *O. schmidti*, and *O. xiangchengensis* (vs. without spot in the latter).

*Oreolalax longmenmontis* sp. nov. most resembles *O. popei* in morphology and is also potentially sympatric with it. The new species could be distinguished from *O. popei* by a combination of following characters: comparatively small body size (mean male SVL 56.8 mm vs. 64.4 mm in *O. popei*), head wider than long (vs. head longer than wide in *O. popei*), tibio-tarsal articulation reaching beyond nostril when leg stretched forward (vs. just reaching the anterior angle of eye in *O. popei*), forelimb long (mean male LAL/SVL ratio 51.89% vs. 46.87% in *O. popei*), hindlimb long (mean male HLL/SVL ratio 182.05% vs. 172.02% in *O. popei*, and mean male TFL/SVL ratio 77.64% vs. 73.21% in *O. popei*), and short IML (4.46% of SVL vs. 5.03% *O. popei*).

*Oreolalax longmenmontis* is genetically closer to *O. nanjiangensis* and *O. chuanbeiensis*. The new species distinctly differs from *O. chuanbeiensis* by the following characters: broader head (vs. head wide almost equal to long in the latter), lacking spines on lip margin in male (vs. visible in male in the latter), spiny patches on chest small with thick sparse spines in male (vs. large with fine spines in the latter), nuptial spines thick sparse on fingers (vs. thin and fine in the latter), toe webbing at base (vs. toe IV 1/3 webbed in the latter), tibio-tarsal articulation reaching beyond nostril when leg stretched forward (vs. just reaching the level of eye in the latter), and having significant differences on HDL, HDW, SL,NS, IND, UEW, LW (*p* < 0.05 when comparing with the latter; Table 3). The new species differs from *O. nanjiangensis* by the following characters: broader head (vs. head wide almost equal to long in the latter), belly with marble spots (vs. without spot in the latter), tibio-tarsal articulation reaching beyond nostril when leg stretched forward (vs. just reaching the level of eye in the latter), interorbital region with dark triangular pattern (vs. without in the latter), comparatively long body size (mean male SVL 56.8 vs. 53.7 in the latter), forelimb long (mean male LAL/SVL ratio 51.9% vs. 48.9% in the latter), and hindlimb long (mean male HLL/SVL ratio 182% vs. 172% in the latter, and mean male TFL/SVL ratio 77.6% vs. 72.7% in the latter; Table 3).

#### Distribution and ecology.

**Figure 7.**
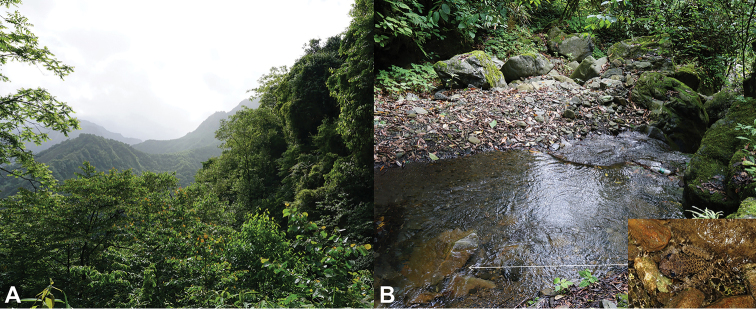
Habitats of *Oreolalax longmenmontis* sp. nov. in the type locality, Sichuan White River National Nature Reserve in Pengzhou City, Sichuan Province, China **A** landscape of montane forests **B** a montane stream with a small pond occupied by toad (*insert*: the holotype CIB20180522001 in life).

*Oreolalax longmenmontis* sp. nov., is currently known only from the type locality, the White River National Nature Reserve, Pengzhou City, Sichuan Prov., China at elevations of 1300–1450 m. The new species inhabits subtropical evergreen broad-leaved forests and is frequently found near the ponds in the montane streams (Fig. 7). The breeding season is currently uncertain. Three sympatric amphibian species, i.e., *Amolops chunganensis* (Pope, 1929), *Odorrana margaratae* (Liu, 1950), and *Quasipaa boulengeri* (Günther, 1889), were found in the same habitat.

#### Etymology.

The specific epithet *longmenmontis* refer to the type locality of the species, the central part of the Longmen Mountains, Pengzhou City of Sichuan Prov., China. We propose the common name “Longmen Mountains toothed toad” (English) and “long men shan chi chan” (Chinese).

## Discussion

Superficial similarities in morphology between related species of the genus *Oreolalax* easily lead to misleading classifications in the field. Although *Oreolalax longmenmontis* sp. nov. resembles *O. popei*, our detailed comparisons can identify them by many morphological characters. A previous study regarded one sample from Pengzhou City, Sichuan Prov., China as *O. popei* (Fu et al. 2007), but which is phylogenetically nested with samples of *Oreolalax longmenmontis* sp. nov. This indicated that the populations from localities near Pengzhou City were probably the new species, such as populations from Maoxian Co. and Dujiangyan City which had been recognized as *O. popei* (Fei et al. 2009, 2012, 2016). These localities, all in the central part of Longmen Mountains, are separated from the type locality of *O. popei*, the Jiajin Mountains in Baoxing Co., Sichuan Prov., by a straight-line distance of 150 km. Future investigation should focus on population composition and exact distributional ranges based on both detailed morphological comparisons and molecular phylogenetic data.

As noted above, basal relationships between major clades of the genus *Oreolalax* were not resolved in all phylogenetic studies (Fu et al. 2007; this study). This was possibly due to the relatively lower number of mutations in 12S and 16S gene sequences and/or historically tachytelic evolution for the basal relationships in the genus. We need more suitable genes or genomic information to resolve systematic profiles in these toads. However, the molecular phylogenetic framework of the genus (Fu et al. 2007; this study) could confirm that the four species groups in *Oreolalax* classified based on morphology by Fei et al. (2005) were all not monophyletic groups.

Unexpectedly, in many and detailed surveys in different seasons, we only found three adult individuals of *Oreolalax longmenmontis* sp. nov. in the White River National Nature Reserve. Obviously, the population, especially the adult population of the species, is extraordinarily small although its tadpole population seems to be rich. It is urgent for us to conduct investigation on its population status because the species suffers from disturbances from tourism, increasingly severe weather, and habitat loss due to intensifying human activities.

## Supplementary Material

XML Treatment for Oreolalax longmenmontis

## Supplementary materials

Supplementary material 1 Table S1 Explanation note Measurements of the *Oreolalax longmenmontis* sp. nov. and its three relatives. See abbreviations for characters in the Materials and methods section. https://binary.pensoft.net/file/402668This dataset is made available under the Open Database License (http://opendatacommons.org/licenses/odbl/1.0/). The Open Database License (ODbL) is a license agreement intended to allow users to freely share, modify, and use this Dataset while maintaining this same freedom for others, provided that the original source and author(s) are credited.Yinmeng Hou, Shengchao Shi, Daming Hu, Yue Deng, Jianping Jiang, Feng Xie, Bin Wang

Supplementary material 2 Table S2 Explanation note Measurements of the *Oreolalax longmenmontis* sp. nov. tadpoles. See abbreviations for characters in the Materials and methods section. https://binary.pensoft.net/file/402669This dataset is made available under the Open Database License (http://opendatacommons.org/licenses/odbl/1.0/). The Open Database License (ODbL) is a license agreement intended to allow users to freely share, modify, and use this Dataset while maintaining this same freedom for others, provided that the original source and author(s) are credited.Yinmeng Hou, Shengchao Shi, Daming Hu, Yue Deng, Jianping Jiang, Feng Xie, Bin Wang

Supplementary material 3 Table S3 Explanation note Examined specimens in this study. https://binary.pensoft.net/file/402670This dataset is made available under the Open Database License (http://opendatacommons.org/licenses/odbl/1.0/). The Open Database License (ODbL) is a license agreement intended to allow users to freely share, modify, and use this Dataset while maintaining this same freedom for others, provided that the original source and author(s) are credited.Yinmeng Hou, Shengchao Shi, Daming Hu, Yue Deng, Jianping Jiang, Feng Xie, Bin Wang
